# A State-of-Art Review of the Vicious Circle of Sleep Disorders, Diabetes and Neurodegeneration Involving Metabolism and Microbiota Alterations

**DOI:** 10.3390/ijms241310615

**Published:** 2023-06-25

**Authors:** Salvatore Versace, Gaia Pellitteri, Roberto Sperotto, Sara Tartaglia, Andrea Da Porto, Cristiana Catena, Gian Luigi Gigli, Alessandro Cavarape, Mariarosaria Valente

**Affiliations:** 1 Clinical Neurology, Udine University Hospital, Piazza Santa Maria della Misericordia, 15, 33100 Udine, Italymariarosaria.valente@uniud.it (M.V.); 2 Department of Medicine (DAME), University of Udine, 33100 Udine, Italyalessandro.cavarape@uniud.it (A.C.); 3 Internal Medicine, Udine University Hospital, 33100 Udine, Italy

**Keywords:** sleep disorders, inflammation, insulin resistance, diabetes, neurodegeneration, cognition, Alzheimer’s disease, gut microbiota, brain–gut axis

## Abstract

In the context of neurodegenerative disorders, cognitive decline is frequently reported in older population. Recently, numerous metabolic pathways have been implicated in neurodegeneration, including signaling disruption of insulin and other glucose-regulating hormones. In fact, Alzheimer’s disease has now been considered as “type-3 diabetes”. In this review, we tried to clarify the role of sleep impairment as the third major player in the complex relationship between metabolic and neurodegenerative diseases. Altered sleep may trigger or perpetuate these vicious mechanisms, leading to the development of both dementia and type 2 diabetes mellitus. Finally, we analyzed these reciprocal interactions considering the emerging role of the gut microbiota in modulating the same processes. Conditions of dysbiosis have been linked to circadian rhythm disruption, metabolic alterations, and release of neurotoxic products, all contributing to neurodegeneration. In a future prospective, gut microbiota could provide a major contribution in explaining the tangled relationship between sleep disorders, dementia and diabetes.

## 1. Introduction

Alzheimer’s disease (AD) and other dementias are a major global health challenge of our century, having a massive impact in terms of size, costs, and social burden. The scientific community worldwide is working to better understand the causes and mechanisms of neurodegeneration as a fundamental step to develop effective disease-modifying therapies for each form of dementia [1].

Sleep is a vital physiological function for humans. Sleep disorders are highly heterogeneous, and abnormal sleep can be the result of alterations in sleep quantity, quality, and structure. Disturbed sleep is very common, especially among older people, and the most frequent conditions are insomnia and sleep-related breathing disorders [2].

The relationship between cognitive and sleep impairment is well established [3]. Almost 60% of patients with any cognitive decline have shown an increased prevalence of different sleep disorders 60% [4]. Conversely, patients with sleep disorders, insomnia and sleep-disordered breathing in particular, have been found to have an 1.19-fold higher risk of developing dementia [5]. Insomnia alone is a risk factor for AD and it causes a faster progression of dementia [6]. Similarly, reduced rapid eye movement (REM) sleep and increased REM sleep latency are both associated with a higher risk of dementia [7]. Like insomnia, excessive daytime sleepiness (EDS) is also related to cognitive decline [8]. Even in the absence of a manifest insomnia disorder, nocturnal sleep of patients with EDS is often superficial and fragmented, with reduced restorative properties. Many sleep disorders other than insomnia can cause EDS in the elderly, including sleep-related movement and breathing disorders [9]. EDS has been independently associated with a diagnosis of dementia, and higher levels of daytime somnolence with lower cognitive performances [10].

Sleep alterations and cognitive impairment share several common structures and pathways. Among them, (i) the neurodegenerative process may involve the suprachiasmatic nucleus (SCN), facilitating sleep alterations in patients with dementia [11]; (ii) sleep fragmentation can affect the clearance of neurotoxic oligomers by decreasing the activity of the glymphatic system in the brain [12]; and (iii) sleep deprivation can trigger cellular and molecular signaling of inflammation, thus inducing cellular aging in response to DNA damage [13].

Both insomnia disorder and EDS predict long-term mortality in the elderly, as these conditions can alter different human systems producing metabolic, endocrine, and immune dysregulation [10,14].

The objective of this review is to focus on these mechanisms, with particular emphasis on how sleep disorders can trigger neurodegeneration by disrupting physiological metabolic pathways, while also impairing the gut microbiota homeostasis.

## 2. Methods

We conducted a narrative state-of-the-art review of PubMed/Medline library, using the following terms in different combinations: “sleep disorder”, “inflammation”, “insulin resistance”, “diabetes”, “neurodegeneration”, “dementia”, “Alzheimer’s disease”, “microbiota”, “brain-gut Axis”. We assessed the contents of both research articles and reviews, excluding gray literature and non-English literature.

Results were evaluated and organized on the basis of the main following associations: “dementia and sleep disorders”, “dementia and metabolic alterations”, “sleep disorders and metabolic alterations”, with particular deepening of common pathophysiologic mechanisms.

In the discussion section, we attempted to integrate the available information in each field to highlight the overlapping pathways between sleep disorders, neurodegeneration, and metabolic alterations, including disruption of gut microbiota homeostasis. We finally suggested possible insights for future research and new therapeutic development.

## 3. Results

### 3.1. Dementia and Sleep: Common Mechanisms

All sleep disorders, including insomnia, sleep-related breathing disorders, excessive daytime sleepiness, circadian rhythm sleep–wake disorders, some parasomnias, and sleep-related movement disorders have been associated with an increased incidence of all-cause dementia [5].

Sleep has a fundamental restorative role for the central nervous system, also playing a crucial part on memory consolidation [12,15]. The brain, in fact, lacks a conventional lymphatic system, but a comparable function is covered by the glymphatic system (GS) [3]. GS is a para-vascular drainage mechanism mediated by the glial cells, which facilitates the clearance of interstitial fluid metabolites in the brain [16]. A convective exchange of cerebrospinal fluid (CSF) and interstitial fluid (ISF) takes place at the level of cerebral vasculature, then the ISF is finally drained to venous circulation [12]. Importantly, metabolic waste products of neural activity and neurotoxic β-amyloid (Aβ), α-synuclein (α-syn) and phosphorylated tau (pTau) aggregates, can be found in the effluent ISF [15]. Slow wave sleep in particular seems to augment the clearance of toxic biomolecules by shrinking glial cells with expanded interstitial space; the result is a greater convective exchange between CSF and ISF, eliminating Aβ during non-REM sleep twice as fast as in wakefulness [12,17]. In murine models, sleep or anesthesia can increase the interstitial space by 60% [15]. In contrast, chronic sleep alteration and insomnia reduce GS activity and facilitate Aβ accumulation [18]. In humans, an inverse relationship between self-reported sleep quality and Aβ deposition has been found. Patients with shorter sleep duration and worse sleep quality have shown a greater Aβ burden measured via positron emission tomography (PET)-amyloid [19]. Moreover, higher neuronal activity has been associated with increased Aβ concentrations in the cerebral interstitial fluid [20]; insomnia notably enhances synaptic and metabolic neuronal activity, that means accelerating Aβ production and aggregation [21].

Another possible common mechanism between sleep disorders and dementia risk involves the activation of the systemic inflammatory response [13]. Different experimental methods, including acute/short and chronic sleep deprivation, seem to stimulate the cellular inflammatory pathways to varying degrees. Previous studies evaluating the relationship between inflammation and impaired sleep have found higher c-reactive protein, interleukin-6 (IL-6) and tumor necrosis factor α (TNFα) levels in poor sleepers [22]. Short sleep duration and sleep fragmentation assessed with objective neurophysiological studies have been associated with higher levels of inflammation [23]. Interestingly, the effect of sleep disruption on inflammation could be compared to other lifestyle-related risk factors, such as sedentary lifestyle or obesity, which are once again risk factors for dementia [22].

Neuroinflammation is then propagated by activation of astrocytes and microglia, offering a major contribution to neurodegeneration [11]. After just one week of sleep restriction, pro-inflammatory cytokines IL-6 and TNFα increase, so even a short sleep loss can impair the performances during the psychomotor vigilance test [24]. Chronic sleep restriction enhances brain interleukin-1β (IL-1β) and TNFα expression, while reducing mRNA levels of brain-derived neurotrophic factor (BDNF); this also contributes to neurocognitive detriment [25]. These inflammatory cytokines contribute to Aβ production and accumulation of a vicious cycle: Aβ plaques can activate astrocytes to secrete the same pro-inflammatory cytokines (TNFα, IL-1β), thereby amplifying the neurodegeneration [26,27].

### 3.2. Dementia and Metabolism: Common Mechanisms

#### 3.2.1. Insulin Resistance and Genetic Variability

In recent years, sporadic AD has been increasingly considered as a metabolic disorder, characterized by brain insulin resistance and impaired glucose metabolism, which is the main energetic source for neurons [28,29]. Evidence of common characteristics between age-related dementia and insulin resistance and diabetes lead to the definition of AD as the “type-3 diabetes” [30]. The brain has a limited storage capacity for glucose, and brain function deteriorates rapidly when the glucose supply is reduced. There is compelling evidence that glucose metabolism in the brain begins to decrease more than 10 years before the occurrence of cognitive symptoms, suggesting that metabolic changes in the brain system are closely related to AD onset [31].

If the brain was traditionally considered insulin-independent, today we know that insulin receptors (IRs) are expressed in the central nervous system. Thus, insulin do affect the uptake of glucose into neurons and energy metabolism in the brain [32]. Neuronal glucose uptake is mainly regulated through glucose transporter 3 (GLUT-3), which is opened by the depolarization of N-methyl-D-aspartate (NMDA) receptors. In fact, IRs trigger intracellular signaling with a substantial role in maintaining brain functions, such as release and re-uptake of catecholamines, ion-channel trafficking, γ-Aminobutyric acid (GABA) and glutamate receptors membrane trafficking. Through different pathways, insulin acts as a growth factor and promotes neuronal metabolism and survival, synaptic density, and neural connectivity, thereby influencing cognitive processes [32].

Several molecular functions are related to insulin resistance in the brain, namely increased activity of pro-inflammatory cytokines, reactive oxygens species, and dysfunction of glucose transporter 4 (GLUT-4) [33]. An impaired insulin signaling could then manifest as type 2 diabetes mellitus (T2DM) at the peripheral level and as AD at the central level. In the brain, impairment of insulin signaling through insulin resistance cause neuronal dysfunction, due to deficits in energy metabolism [27,34]. A large number of studies in the last two decades suggest that central insulin resistance and cognitive decline in AD are associated with changes in the neuronal insulin/IR signal transduction cascade, insulin grow factor 1 (IGF-1) resistance and insulin receptor substrate 1 (IRS-1) dysregulation [35,36].

Insulin resistance increases Aβ production in the brain stimulating β-site amyloid precursor protein cleaving enzyme 1 (BACE1) and glycogen synthase kinase 3 (GSK-3) activity; it also impairs normal brain function by reducing insulin signal cascade, reduces the inhibition of Aβ toxicity, and accelerates tau hyperphosphorylation [37]. Hyperinsulinemia could increase Aβ production and release, thereby also reducing its degradation [38]. In particular, insulin competes with Aβ for the activity of insulin-degrading enzyme, facilitating Aβ accumulation [38].

The apolipoprotein E4 (*APOE4*) gene variant is the strongest genetic risk factor for AD, and it has been demonstrated that ApoE4 interferes with IR signaling, suggesting a role for ApoE4 in association with insulin resistance in the pathogenesis of AD [39].

#### 3.2.2. Hormonal Disruption

In addition to insulin resistance, alterations in other important hormones related to glucose metabolism also contribute to neurodegeneration.

First, it is the growth hormone (GH) and IGF-1 axis [40]. IGF-1 is the central mediator for GH activity and promotes neuroprotective effects; lower serum levels of IGF-1, which are common in the elderly, are associated with cognitive decline [41].

Second, it is the glucagon-like peptide 1 (GLP-1). GLP-1 is an incretin released by intestinal L cells, promoting glucose-dependent insulin secretion. GLP-1 also produces various non-glycemic effects through widespread GLP-1 receptors, in particular controlling gastrointestinal motility and delaying gastric emptying. In addition to L-cells’ endogenous rhythm, other factors including dietary composition, obesity, prolonged light exposure, sleep disturbances and intestinal flora disorders can influence the rhythmic secretion of GLP-1. Disruption of GLP-1 rhythm leads to a derangement of the corresponding physiological insulin secretion rhythm [42]. GLP-1 does not cross the blood–brain barrier, hence only GLP-1 expressed in the nucleus accumbens acts on the cerebral GLP-1 receptors suppressing appetite. Furthermore, GLP-1 reduces the levels of endogenous Aβ deposition in the brain, and prevents tau hyperphosphorylation. On the basis of this possible neuroprotective effect, GLP1 is currently under investigation as a potential disease-modifying treatment for AD; it could promote cell differentiation, neurogenesis, synaptic plasticity, attenuating oxidative stress and mitochondrial dysfunction, inhibiting neuronal apoptosis and neurotoxicity [43].

GLP-1 analogs are approved as a first-line therapy for T2DM and obesity. These molecules work with various mechanisms, including restoration of GLP-1 physiological secretion rhythm, amelioration of insulin secretion, anti-inflammatory properties, regulation of the intestinal flora, appetite suppression and weight reduction; interestingly these drugs have also shown positive effects on cognitive function and mood. GLP-1 receptor agonists (GLP-1Ras) have anti-inflammatory effects in the central nervous system, contrasting neuroinflammation in AD models, thereby improving cognitive dysfunctions. Furthermore, GLP-1Ras might improve cognitive function and memory in humans, directly enhancing the mechanisms of anti-Aβ aggregation/deposition and anti-tau hyperphosphorylation effects [43].

Preclinical studies have suggested the potential role of newer glucose-lowering drugs, including dipeptidyl peptidase (DPP-4) inhibitors, GLP-1 RAs and sodium glucose co-transporter-2 (SGLT-2) inhibitors in protecting humans against cognitive decline. However, population studies aiming to demonstrate cognitive benefits of antidiabetic agents have shown contrasting results, also due to a large heterogeneity of design, and should be interpreted with caution [44].

#### 3.2.3. Gut Microbiota 

There is a growing emphasis on the relevance of the gut microbiota (GM) and its composition for human health; various states of dysbiosis may contribute to different pathological processes, including neurodegeneration [45,46]. Most of the research investigating the relationship between microbiota and dementia has been conducted on animal models, but there is some evidence on humans with different causes of dementia as well [47]. It has been suggested that specific GM alterations may favor more than one predisposing condition to neurodegeneration, such as T2DM and obesity [47,48].

The first pioneer who hypothesized a connection between microbiota and dementia was Alois Alzheimer himself in the late 1800s [49]. So far, many predisposing and protective bacteria have been identified [50]. Li et al. tested fecal and blood samples of patients with mild cognitive impairment (MCI), AD and healthy subjects, demonstrating strong differences in microbiota composition of MCI and AD patients compared to healthy subjects [51]. Cattaneo et al. [52] compared amyloid-positive with amyloid-negative patients, observing a relative abundance of Escherichia/Shigella in the first group, with higher circulation of pro-inflammatory cytokines, namely IL-6, IL-1β, chemokine (C-X-C motif) ligand 2 (CXCL2), and NOD- LRR- and pyrin domain-containing protein 3 (NLRP3). Vogt et al. [51] found an increase in Bacteroidetes and lower amounts of Firmicutes and bifidobacteria in AD patients. These results were further confirmed by Zhuang et al. [53].

The mechanisms through which GM may affect neurocognition are not well understood. Several theories have been proposed to date, the most relevant can be summarized as follows. (1) An overexpression of pathobionts may predispose to the intestinal barrier disruption, leading to an increase in circulating bacteria and toxins. In fact, higher levels of Gram-negative bacterial lipopolysaccharides (LPS) were detected in blood samples of patients with a “leaky gut” barrier [54]. Additionally, two to three times increased levels of LPS were found in post-mortem brain samples of AD patients, mainly located in the hippocampal and temporal neocortex [55]. LPS deposition, together with bacterial amyloid-like substances [54,56], may cause macrophages and T-cell activation with following release of pro-inflammatory cytokines and reactive oxygen species (ROS) [57,58,59,60]. Pathobionts themselves can also produce Aβ-like metabolites able to cross-seeding with human Aβ; resulting amyloid deposition in the brain stimulates an inflammatory response by the local microglia, with plaques formation and neuronal loss [58,59,60,61,62]. (2) Many other bacterial toxins may influence Aβ plaques deposition as well, like saxitoxin, anatoxin-alfa or beta-N-methylamino-alanine [63]. On the other hand, short-chain fatty acids (SCFAs), especially butyrate, and histamine may have a neuroprotective role [64,65]. (3) It is possible that the lack of probiotic strains may affect the secretion of neurotrophic factors, such as BDNF, NMDA and GABA [66].

### 3.3. Sleep and Metabolism: Common Mechanisms

#### 3.3.1. OSAS, Insulin Resistance, and Hormonal Disruption

Poor sleep has been associated with both central and peripheral insulin resistance, facilitating impaired glucose metabolism and neurodegeneration [27,67]. Indeed, acute and chronic sleep deprivation may alter glucose and insulin levels. It also interferes with appetite regulation, leading to weight gain, thereby indirectly contributing to the phenomenon [68]. Patients with obstructive sleep apnea syndrome (OSAS) have a concrete risk to fall into a vicious cycle of sleep fragmentation, worse metabolic control and weight gain, further exacerbating nocturnal apnea and sleep quality. Hypocretins, also known as orexins, are important hypothalamic hormones promoting wakefulness and food intake [69]. Sleep deprivation increases their release, thus promoting their interaction with other neuro-hormonal pathways. At the peripheral level, orexins lead to lower leptin and higher ghrelin concentrations, with the final effect of stimulating appetite [68]. Increased abdominal fat and soft tissues surrounding the upper airways contribute to OSAS development [70]. The syndrome itself generates a feedforward cascade of negative events, due to sleep loss, sleep fragmentation and hypoxia, worsening the pre-existent metabolic alterations, specifically insulin resistance [68].

Orexin deficiency is the pathological basis of narcolepsy type 1 (previously narcolepsy with cataplexy) [71]. Interestingly, in addition to the typical pentad of symptoms (i.e., EDS with sleep attacks, cataplexy, sleep paralysis, hypnagogic hallucinations, and fragmented nocturnal sleep), those patients often present with a variety of metabolic and endocrine alterations, suggesting a more extensive hypothalamic involvement. Increased frequency of overweight and obesity conditions is observed in children with narcolepsy, and diabetes in narcoleptic adults [72]. Despite these associations, a potential relationship between narcolepsy and dementia has never been investigated so far.

Sleep disorders promote insulin resistance, also disrupting other hormonal pathways, particularly GH/IGF-1 and GLP-1 axis. Slow wave sleep normally increases GH/IGF-1 axis activity [73]. Conversely, after five days of sleep deprivation, IGF-1 was found to be decreased in human peripheral blood, together with increased pro-inflammatory factors [74]. Furthermore, GLP-1 intestinal secretion is regulated by numerous factors, including sleep. Circadian regulation of intestinal L-cells activity is highly sensitive to sleep alterations. In fact, there is clear evidence suggesting that short sleep duration impairs normal fluctuations in GLP-1 levels and nutrient-induced insulin response. Disruption of GLP-1 rhythm then leads to a derangement of the physiological insulin secretion rhythm and impaired glucose tolerance [42].

#### 3.3.2. Circadian Rhythm and Clock Genes

Human metabolism is closely synchronized with circadian sleep–wake cycle and mealtimes. Circadian rhythms (CR) actually play a key role in the mutual relationship between sleep alterations and the disruption of human metabolic pathways. CR could be defined as the internal process that synchronize behavioral and biochemical processes with the external day/night cycle [75]. The SCN acts as the central clock, maintaining an endogenous circadian activity and regulating the peripheral clocks of different organs (e.g., intestine, liver, and pancreas) [76]. In addition to integrating SCN signals, these peripheral clocks are influenced by environmental and behavioral synchronizers (also known as *zeitgebers*), such as light, feeding, sleep and life schedules [77]. At the molecular level, intracellular circadian clock is maintained by the so-called “clock” genes; they mostly encode for transcription factors which are able to downregulate their own expression in favor of other clock genes [76,77]. Among them, circadian locomotor output cycles protein kaput (*CLOCK*) and brain and muscle ARNT-like protein (*BMAL1*) genes on one side, and period (*PER*) and cryptochrome (*CRY*) genes on the other, are reciprocally involved in a fine oscillating feedback mechanism [77,78]. Gene products are assembled into large heterotypic complexes, mutually interacting and regulating their expression [77,78].

Clock transcription factors can influence metabolism in several ways, impacting bile acid production, lipogenesis, osteogenesis, glucose and energy homeostasis [75]. In mouse models, disruption of light–dark cycle obtained with 24 h light exposure can reduce glucose-stimulated insulin secretion. This result is explained by an altered function of molecular oscillators located in pancreatic islets, which are essential for proper β-cell function [79].

Based on the current literature, the disruption of CR caused by work-shift schedules and irregular eating patterns is associated with an increased risk of T2DM by way of impaired glucose metabolism and insulin resistance. Night-shift workers constitute the most representative human model, demonstrating the association between CR alterations and increased risk of T2DM [80]. Higher frequency of rotating shifts has been associated with higher diabetes risk, with a dose-dependent effect [81]. Indeed, decreased β-cell function and increased postprandial glucose concentration have been observed during night shifts, resembling the pattern of patients with impaired glucose tolerance or diabetes [82].

#### 3.3.3. Gut Microbiota

As previously reported, many exogenous factors may influence CR with increasing risk of T2DM. However, less is known about the role of endogenous factors and their relationship with CR in predisposing patients to diabetes. Among them, the GM is increasingly recognized as a major player.

GM and CR are engaged in a bidirectional relationship. The intestine acts as a peripheral circadian clock; it is synchronized with the hypothalamic SCN (i.e., the central circadian clock) but also exhibits its own oscillations [83]. The evidence on GM’s ability to regulate or modify host CR comes from animal models. In germ-free mice, chrono-biological cues from gut bacterial flora (i.e., SCFAs) are inevitably absent; despite normal light and dark signals, these experimental models have shown a downregulation in circadian gene expression in central and peripheral tissues [84].

Both in humans and animal models, the GM itself shows diurnal oscillations in composition and function, which seems to be influenced by host feeding schedule. GM dysbiosis is registered in patients with CR alterations, due to specific sleep disorders, social habits, shift work, or jet lag disorder. As a matter of fact, these are subjects with increased metabolic alterations and higher obesity and T2DM incidence [85]. Interestingly, microbiota samples collected from patients with jet lag disorder have been found with higher expression of Firmicutes, a bacterial phylum associated with higher risk of obesity and metabolic disorders in humans. In addition, there is some evidence in mice that fecal transplantation may carry this propensity to metabolic disorders, suggesting a possible role of dysbiosis in contributing to altered host metabolism [86].

GM can regulate daily cycles epigenetically, inducing rhythmic expression of histone deacetylase 3 (HDAC3) in epithelial cells of the small intestine. HDAC3 plays a role in various nutrient uptake (aromatic amino acids, inositol, and riboflavin) and intestinal lipid absorption, by modulating the expression of lipid metabolism genes. Experimental mice lacking HDAC3 have increased glucose tolerance, decreased insulin resistance and lower fat accumulation. Therefore, we could assume that CR disorders may alter the GM with subsequent disruption of the HDAC3 pathway, thereby contributing to explaining obesity and T2DM in patients with circadian sleep alterations [87].

GM also influences body composition through the circadian transcription of nuclear factor interleukin-3 (NFIL3). This protein mediates the expression of the circadian lipid metabolic program, regulating lipid absorption and transport in intestinal epithelial cells [88]. Undeniably, NFIL3 could represent another molecular link among microbiota, circadian clock, and host metabolism.

Finally, the GM regulates the host circadian clock by producing SCFAs, which modulate hepatic expression of clock genes [77]. One of the mediators of SCFAs is the short-chain fatty acid receptor GPR43 (G protein-coupled receptor 43), which works as a sensor for excessive dietary energy. When activated, GPR43 suppresses insulin signaling in adipocytes, inhibiting fat accumulation in adipose tissue and promoting lipids and glucose metabolism in other tissues [89]. To confirm this, knock-out mice for GPR43 are obese on a normal diet, whereas mice overexpressing GPR43 in adipose tissue remain lean even on high-fat diet [89].

All this evidence suggests that GM is in a close relationship with the circadian clock, and that dysbiosis could significantly contribute to the development of metabolic disorders, including insulin resistance, glucose intolerance, T2DM and obesity [77].

## 4. Discussion

**Figure 1:**
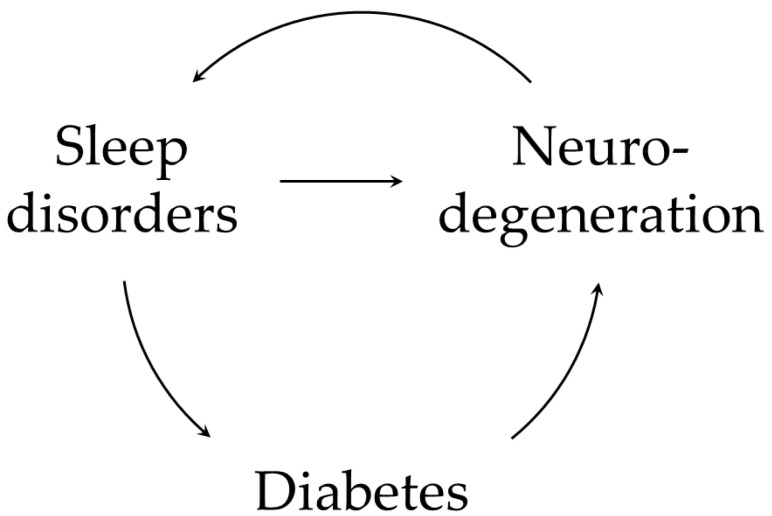
A summary of possible interactions between sleep disorders, diabetes, and neurodegeneration. Sleep impairment can lead to neurodegeneration and cognitive decline; part of this process is mediated by metabolic alterations [90]. Sleep alterations contributes to amyloid accumulation by impairing glymphatic system function and favoring the activation of systemic inflammatory response. Moreover, disturbed sleep dysregulates crucial hormones involved in glucose homeostasis (i.e., insulin, GH/IGF-1 axis and GLP-1), predisposing to both T2DM and AD. These mechanisms can enter a vicious cycle and build on each other. Neuroinflammation propagates via the activation of astrocytes and microglia; Aβ plaques themselves can activate astrocytes to release pro-inflammatory cytokines. Neurodegeneration also involves crucial structures for sleep physiology (e.g., hypothalamic SCN) [11] perpetuating and worsening sleep alterations, with further amyloid deposition.

The main purpose of this review was to investigate the potential relationship between sleep disorders and neurodegeneration, particularly focusing on the possible intermediary role of disrupted metabolism and GM alterations (Figure 1). Thanks to the knowledge acquired in the last decades, AD is now also considered as a metabolic disease with mechanisms of impaired brain glucose metabolism [28,29]. Some aspects of these complex interactions have already been investigated, although an extensive picture of all the implicated pathways and associated causative mechanism was still missing in the literature. Holingue C. et al. [90] have previously proposed some models to explain how sleep, cognitive decline and metabolism can interact. In one of their hypotheses, sleep disturbance was designed as the causative factor leading to cognitive impairment through alteration of metabolic pathways.

**Figure 2:**
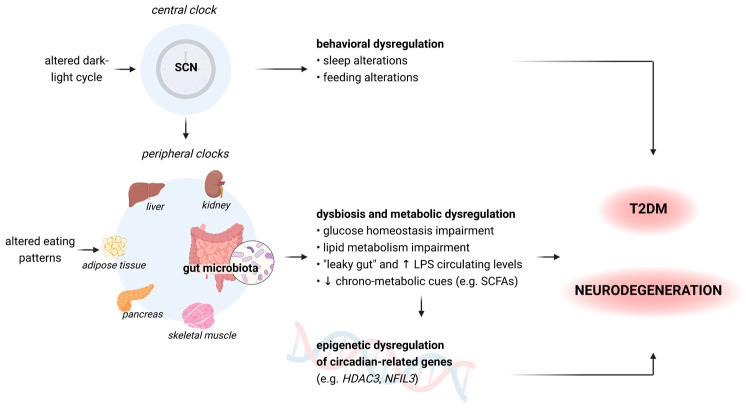
A summary of how central and peripheral clocks alterations can lead to a consequential behavioral and metabolic dysregulation, thus contributing to the development of diabetes and dementia [75,77,86]. The hypothalamic SCN acts as the central clock, modulating the intrinsic oscillating activity of peripheral clocks. SCN: suprachiasmatic nucleus; LPS: lipopolysaccharides; SCFAs: short-chain fatty acids: *HDAC3*: histone deacetylase 3 gene; *NFIL3*: nuclear factor interleukin 3 gene. Figure 2 was created with BioRender.com, accessed on 15 June 2023.

To the best of our knowledge, this is the first review that analyzes these pathways in light of the increasing evidence on gut microbiota and its emerging role in all these processes (Figure 2).

Firstly, sleep alterations seem to disrupt the regulation of all crucial hormones in glucose homeostasis, notably insulin, GH/IGF-1 axis and GLP-1. The same hormones are the ones implicated in both T2DM and AD development. On the other hand, the rhythmic secretion of endogenous GLP-1 can be restored, and glucose metabolism and insulin resistance can be improved by treating sleep disorders or ameliorating sleep quality. In summary, disturbed sleep could induce insulin and IGF-1 brain and peripheral resistance, facilitating both impaired glucose metabolism and neurodegeneration [27,67].

Secondly, peripheral and central inflammation related to sleep loss may play a role in cognitive impairment [40]. As reported above, acute and chronic sleep restriction increase pro-inflammatory cytokines levels, while reducing anti-inflammatory mediators. Inflammatory cytokines contribute to a vicious cycle of Aβ production and accumulation [26]. In diabetic patients, peripheral insulin resistance also increases toxic lipids products, which cross the blood–brain barrier, and contribute to neuroinflammation [34].

Lastly, individual sleep status exerts its metabolic effect by influencing GM composition, which in turn can alter insulin metabolism and cognitive functions. For example, a healthy microbial gut environment is necessary for the rhythmic secretion of GLP-1 [42]. Sleep loss can promote Aβ plaques deposition via the GM. Sleep loss, as a biological stressor, can induce hypothalamus–pituitary–adrenal (HPA) axis activation, following with the release of catecholamines and glucocorticoids. These molecules and pathways, for their part, may affect the general composition of the GM [91] and the intestinal barrier integrity. It results in a more vulnerable “leaky gut”, leaving increased access of bacteria and metabolites in blood circulation. A typical sleep-induced GM dysbiosis include proliferation of pathobionts, Erysiopelotrichaceae and Enterobacteriaceae, and decrease in SCFAs-producing bacteria [50]. On a speculative plan, this process could lead to blood–brain barrier disruption, neuroinflammation, increased concentration of ROS, lower Aβ clearance and Aβ plaques deposition [92]. The interaction between HPA axis and microbiota is reciprocal, and the fact that GM varies HPA set-point, leads to changes in its reactivity [93,94]. After just two nights of sleep restriction, gut microbiota alterations are similar to those registered in patients with obesity T2DM: increased Firmicutes to Bacteroidetes ratio, together with increased Coriobacteriaceae and Erysipelotrichaceae, and reduced Tenericutes [95].

GM can also modulate the inflammatory response causing cognitive decline in sleep deprivation (SD) models. Fecal transplants from SD humans to germ-free mice promoted proinflammatory phenotypes in the dorsal hippocampus and medial prefrontal cortex. Colonization with SD-associated microbiomes induces a condition of gut leakage with higher circulation of LPS and other bacterial toxins in mouse models, resulting in a systemic inflammatory response. In particular, increased levels of pro-inflammatory cytokines (such as TNFα, IL-1β, and IL-6), lower levels of interleukin-10 (IL-10) and down-regulation of anti-inflammatory activity have been observed [96]. Opposite results have been found for SCFAs production: lower concentrations of acetate, propionate and butyrate have been registered in stool samples of transplanted mice; lower circulating levels of butyrate were also detected in mice serum [96].

Furthermore, transplanted mice have shown a reduction in cognitive performances, likely due to neuroinflammation induced by the SD-associated microbiota. Although in animal models, some reports suggest that SD consequences could be transmissible from one organism to another by GM transplantation [96].

We also highlighted how night-shift workers constitute the best human model, demonstrating the relationship between CR alterations and the increased risk of T2DM. Ultimately, night-shift work has been associated with an increased incidence of dementia with a dose-dependent effect [97].

In summary, sleep loss seems to trigger and perpetuate metabolic derangements, impairing glucose metabolism and predisposing individuals to develop both diabetes and dementia. Part of these results are mediated by the effect of altered sleep on GM composition, due to their close mutual interactions.

In the future, more in-depth knowledge of the interaction mechanisms between the GM and neurodegeneration will be needed; some interventions, such as as probiotic [98,99,100,101,102,103,104] or antibiotic treatments [105,106,107,108,109], and even fecal microbiota transplantation (FMT), [110,111] could have a therapeutic role in changing the course of AD. There is emerging evidence on the protective effects of GM treatments in animal models. Probiotics, such as Lactobacillus and Bifidobacterium, seem to ameliorate symptoms of AD. Some strains of these bacteria have been tested on mice, with results of decreasing Aβ deposition, inflammatory activity, and cognitive deficits. Similar results may be achieved with FMT [110].

A recent comprehensive review investigated the relationship between dysbiosis, altered inflammatory cytokines profile and microglia in preclinical models of AD, T2DM, and combined AD/T2DM models; the use of pre- and probiotics supporting a healthy GM in those models ameliorated both AD and T2DM pathologies [112].

## 5. Conclusions

Current studies show the association between AD, insulin resistance and T2DM, although common pathophysiologic mechanisms are still not fully elucidated. Dietary habits and lifestyle are risk factors for both diseases, but they also impact GM composition and function. In addition, AD and other dementias are comorbidities associated with sleep disorders, while altered sleep is a risk factor for dementia itself.

It is reasonable to conclude that AD and altered sleep share similar abnormal pathways including GM dysfunction, with possible reciprocal positive or negative interactions. Through bacterial metabolites, the brain–gut axis actually plays a relevant role in the pathogenesis of AD, T2DM, neuroinflammatory processes and sleep disorders.

Based on the available evidence, we can hypothesize that there is a tangled relationship between these conditions, and they are all involved in a self-strengthening vicious circle. In conclusion, we may theorize that basic clinical interventions aiming at improving sleep and GM composition could help to prevent and treat metabolic and neurodegenerative diseases. However, high-quality evidence on such therapeutic approaches is still lacking in the current literature, and thus further research will be needed.

## Data Availability

Data sharing not applicable.
